# Non-steroidol anti-inflammatory drug effect on crypt cell proliferation and apoptosis during initiation of rat colon carcinogenesis.

**DOI:** 10.1038/bjc.1998.93

**Published:** 1998-02

**Authors:** C. J. Barnes, I. L. Cameron, W. E. Hardman, M. Lee

**Affiliations:** Department of Medicine, University of Texas Health Science Center, San Antonio 78284-7878, USA.

## Abstract

**Images:**


*British Journal of Cancer* (1998) **77**(4), 573–580
© 1998 Cancer Research Campaign

### Non-steroidol anti-inflammatory drug effect on crypt cell proliferation and apoptosis during initiation of rat colon carcinogenesis

**CJ Barnes[1], IL Cameron[2], WE Hardman[2] and M Lee[3]**

Departments of [1]Medicine and [2]Cellular & Structural Biology, University of Texas Health Science Center; and [3]Veterans Affairs Medical Center, San Antonio, TX 78284, USA

**Summary** Sustained use of non-steroidal anti-inflammatory drugs (NSAIDs) may prevent colorectal cancer. However, the optimal drug, period of efficacy and mechanism(s) of action are unknown. Experiments were undertaken to determine which of several NSAIDs would modulate colon crypt cell proliferation or apoptosis when given during the initiation phase of 1,2-dimethylhydrazine (DMH)-induced rat colon cancer. Colon crypts located both away from and over an aggregate of lymphoid nodules (ALN) were examined. Rats were injected with aspirin, indomethacin, nabumetone, sodium salicylate, 16,16-dimethyl prostaglandin $E_2$ or saline for 3 days and DMH or DMH vehicle on day 4 of each week for 8 weeks, then killed 3 days after the last DMH injection. At the time of killing, DMH had significantly increased crypt cell proliferation but not apoptosis. There was significantly more cell proliferation and apoptosis in crypts over the ALN than away from the ALN. Aspirin and salicylate increased proliferation and apoptosis in crypts over the ALN. Finally, the distributional peaks of cell proliferation and apoptosis were shifted significantly closer together after DMH. Thus, DMH increases proliferation and alters the distribution of proliferating and apoptotic cells in colon crypts early in carcinogenesis. Aspirin may suppress tumour incidence via salicylate by enhancing apoptosis in carcinogen-initiated cells.

**Keywords:** colon cancer; proliferation; apoptosis; non-steroidal anti-inflammatory drugs; lymphoid nodules; 1,2-dimethylhydrazine

Data from human epidemiological research (Thun, 1994; Giovannucci et al, 1995) and from rodent experimental research (Craven and DeRubertis, 1992; Reddy et al, 1993) have demonstrated both correlative and direct evidence of a role for aspirin and possibly other non-steroidal anti-inflammatory drugs (NSAIDs) in the prevention of colon and rectal cancer morbidity and mortality. However, the optimal NSAID, the dose, the period of efficacy and the mechanism of this putative chemopreventive activity remain to be established (Earnest et al, 1992; Giardiello et al, 1995).

One potential mechanism for this chemopreventive effect lies in the ability of aspirin and other NSAIDs to inhibit prostaglandin production by blocking the activity of one or both of the prostaglandin endoperoxide G/H synthase (PGHS) isozymes, PGHS-1 and PGHS-2 (Meade et al, 1993). PGHS-1 is the constitutive form of the enzyme and is expressed in most tissues, whereas PGHS-2 is induced by mitogenic stimuli in inflammatory situations (Smith and DeWitt, 1996) and is expressed at high levels in colon tumour tissue (Eberhart et al, 1994; Kargman et al, 1995; Dubois et al, 1996). Aspirin covalently and irreversibly modifies the cyclo-oxygenase active site of both PGHS-1 and PGHS-2 by acetylation, whereas other NSAIDs reversibly block PGHS function and thus prostaglandin production via steric hindrance of the cyclo-oxygenase active site (Smith and DeWitt, 1996).

*Received 28 February 1997*
*Revised 12 August 1997*
*Accepted 14 August 1997*

*Correspondence to:* CJ Barnes, Department of Medicine/Division of Gastroenterology, University of Texas Health Science Center, 7703 Floyd Curl Drive, San Antonio, TX 78284-7878 USA

Several lines of evidence implicate prostaglandin (PG) involvement in colorectal cancer (Marnett, 1992). These include: evidence that PGs stimulate proliferation of human colon cancer cell lines in vitro and normal rodent colon epithelial tissue in vivo (Qiao et al, 1995); evidence that progressively increased tissue prostaglandin $E_2$ ($PGE_2$) levels are associated with progression of colon carcinogenesis (Pugh and Thomas, 1994); and a possible immunosuppressive effect of $PGE_2$ (Goodwin, 1981). Thus, NSAID inhibition of intestinal prostaglandin production may have a number of direct chemopreventive activities against the progression of colorectal cancer.

Other potential mechanisms for chemoprevention by NSAIDs include alteration of colon epithelial cell proliferation and/or apoptosis. A tumour is generally considered to be a lesion that arises as a result of an imbalance between cell proliferation and cell death. Increased epithelial cell proliferation is an established risk factor for colon cancer development (Deschner and Maskens, 1982; Wright and Alison, 1984; Cameron et al, 1990). NSAIDs have been shown to reduce cell proliferative parameters in colon crypts (Barnes et al, 1995) and to stall cultured colon adenocarcinoma cells in the $G_0/G_1$ phase of the cell cycle (Shiff et al, 1996). NSAIDs may also restore normal colon mucosal homeostasis in situations of increased proliferation by inducing apoptotic cell death (Pasricha et al, 1995; Shiff et al, 1995; 1996).

This report examines whether NSAIDs, specifically aspirin, indomethacin or nabumetone, would prevent initial preneoplastic changes in colon epithelial cell proliferation or apoptosis when administered only during the initiation phase of 1,2-dimethylhydrazine (DMH)-induced rat colon cancer. Either aspirin and/or its metabolite, salicylic acid, could be the active molecule in colon cancer chemoprevention; thus, this study included both aspirin and sodium salicylate treatment groups. In addition, 16,16-dimethyl-prostaglandin $E_2$ ($dmPGE_2$), a stable $PGE_2$ analogue that has been shown to stimulate proliferation of colonocytes both in vitro and in vivo (Qiao et al, 1995), was tested as a positive control for inhibition of prostaglandin synthesis by NSAIDs. The effects of these drugs on cell proliferation and apoptosis were examined in rat colon crypts located both over the rat distal colon aggregate of lymphoid nodules (ALN, a site of rapid epithelial cell proliferation and of high risk for adenocarcinoma occurrence; Hammann et al, 1992; Cameron et al, 1996) and away from an ALN. Finally, this report demonstrates: (a) the simultaneous use of the cell proliferative and apoptotic indices in colon crypts; and (b) that the distribution of proliferative and apoptotic cells in colon crypts can be used as an early biomarker of neoplastic change.

**Figure 1** Photomicrographs of representative apoptotic figures (arrowheads) found at different points along the crypt column in haematoxylin and eosin-stained 4-μm sections of rat colon tissue. *Bar* = 20 μm

### MATERIALS AND METHODS

### Animals

Male Sprague–Dawley rats were purchased at 2 months of age from Harlan Sprague–Dawley (Indianapolis, IN, USA) 4 weeks before the experiment. Rats had free access to Teklad ML485 Rat Diet (Teklad/HSD, Madison, WI, USA) and water before and throughout these studies. The rats were housed in wire-bottom cages to prevent coprophagy. All experimental procedures described below were approved by the Institutional Animal Care Program of the University of Texas Health Science Center (San Antonio, TX, USA).

### Reagents

The following materials were used in these studies: DMH, acetylsalicylic acid (ASA, aspirin), sodium salicylate, indomethacin, and 16,16-dimethyl-prostaglandin $E_2$ ($dmPGE_2$, a stable $PGE_2$ analogue) were purchased from Sigma (St Louis, MO, USA); nabumetone was a generous gift from SmithKline Beecham, King of Prussia, PA, USA; an antiproliferating cell nuclear antigen (PCNA) monoclonal antibody (PC10 clone) was purchased from Signet (Dedham, MA, USA); and the Apoptag kit (purchased from Oncor, Gaithersburg, MD, USA) was used in the TdT UDP nucleotide end-labelling (TUNEL) assay for fragmented DNA ends. All other chemicals were of the highest grade available and were obtained commercially. DMH vehicle contained 0.9% sodium chloride and 0.18% EDTA, pH 6.5, and all other solutions were prepared and diluted with normal saline (0.9% codium chloride solution, pH 6.5). All solutions were prepared 1 h before injection.

### Experimental protocol

After 4 weeks of acclimation and monitoring, rats were randomly divided into one of six treatment groups ($n$ = 12 per group) and were given a single s.c. injection of either saline (4 ml $kg^{-1}$ body weight), acetylsalicylic acid (aspirin, a non-specific PGHS inhibitor, 50 mg $kg^{-1}$), sodium salicylate (the degradation product of aspirin, 50 mg $kg^{-1}$), indomethacin (a selective PGHS-1 inhibitor, 4 mg $kg^{-1}$), nabumetone (a selective PGHS-2 inhibitor, 100 mg $kg^{-1}$) or $dmPGE_2$ (a major colon prostaglandin, 50 mg $kg^{-1}$) on three successive mornings. Aspirin, indomethacin and nabumetone were given in dosages that have significant anti-inflammatory action and that would significantly inhibit PG biosynthesis (Meade et al, 1993; Laneuville et al, 1994). $DmPGE_2$ was administered at a dose that has been shown to confer mucosal protection against NSAID-induced injury in the gastrointestinal tract (Lee and Feldman, 1992). On the fourth morning, half of the rats in each group ($n$ = 6) were given a s.c. injection of DMH (12 mg base per kg bw) and the other half were injected with DMH vehicle alone (4 ml $kg^{-1}$). This weekly pattern was repeated for 8 weeks according to an established protocol for inducing rat colon cancer (Cameron et al, 1990). At the start of week 9 (i.e. 3 days after the last DMH dose), the rats were anaesthetized and killed by decapitation. Immediately after killing, each colon was resected, rinsed with ice-cold phosphate-buffered saline (PBS, pH 7.4) and examined

© Cancer Research Campaign 1998

macroscopically. A 2-cm length of bowel was flash-frozen in liquid nitrogen and stored at –80°C for radioimmunoassay of cyclo-oxygenase activity, as described previously (Redfern et al, 1987; Lee and Feldman, 1992), using the formation of $PGE_2$ from radiolabelled arachidonic acid as an end point. The remaining distal colon was stretched to remove longitudinal folds and pinned flat, serosa side down, on a piece of cork board. The pinned colons were fixed in 10% buffered formalin for 2 h, then stored in 70% ethanol. Rats that received DMH gained significantly less weight ($P < 0.05$) than the DMH vehicle control rats during this 8-week period (82.8 ± 9.89 and 118 ± 8.11 g ± s.e.m. respectively), but there was no significant difference in weight gain due to the other drug treatments (data not shown).

### Proliferative analyses

Transverse segments of the rat descending colon were excised from approximately 3 cm proximal to the anus through the distal colon ALN (over the distal ALN) and from 6 cm proximal to the anus (away from an ALN) and from approximately 3 cm proximal to the anus through the distal colon ALN (over the ALN), and prepared for histology. Four-μm-thick cross-sections of each paraffin-embedded colon segment were cut, mounted on polylysine-coated glass slides, deparaffinized through a series of xylene and graded ethanol washes and incubated with an anti-PCNA monoclonal antibody using routine immunohistochemical procedures, as previously described (Hardman and Cameron, 1994). The validity of using PCNA immunohistochemistry for assessment of cell proliferation in the colon epithelium has been reported previously (Risio et al, 1993; Bostick et al, 1994). As PCNA exhibits a gradation of nuclear staining in formalin-fixed tissue, strict selection criteria were used in which the number and crypt position of only the most intensely stained cells were recorded.

Crypts were included in the assessment if they spanned from immediately adjacent to the muscularis mucosae to the surface epithelium, with the entire lumen of the crypt visible. Cell proliferation parameters were scored for at least 12 crypts per rat in each colon region as reported previously (Hardman and Cameron,

**Figure 2** Frequency distributions of proliferative cells identified by positive PCNA staining, apoptotic cells identified by strict morphological criteria, and of crypt heights in midaxially sectioned colon crypts from rats which were given eight weekly injections of DMH (-------) or DMH vehicle (———). Data are from crypts located (A) approximately 6cm from the anus or (B) directly over the distal colon ALN, approximately 3cm from the anus. For presentation, distributions have been smoothed over five cell positions along the x-axis. The numerical distributional means are presented in Table 4

© Cancer Research Campaign 1998

**Table 1** Effects of different drug treatments on the mean (± 95% confidence intervals) number of PCNA positively stained cells per midaxial crypt section as scored in crypts located either over or away from the distal aggregate of lymphatic nodules

| Group | DMH[a] | Away ALN[b] | Over ALN |
|---|---|---|---|
| Saline | – | 2.99 ± 1.18 | 6.94 ± 1.89 |
| Saline | + | 6.67 ± 2.52 | 6.09 ± 2.44 |
| Aspirin | + | 3.70 ± 1.69 | [c]16.61 ± 6.55 |
| Salicylate | + | 4.83 ± 2.48 | [c]19.54 ± 4.99 |
| Indomethacin | + | 3.57 ± 0.92 | 10.21 ± 1.50 |
| Nabumetone | + | 6.82 ± 2.35 | 11.48 ± 6.81 |
| dmPGE$_2$ | + | 5.88 ± 2.91 | 11.94 ± 1.43 |

[a](–) DMH vehicle (+) DMH (12 mg base per kg body weight) s.c. once a week for 8 weeks, with six rats per group. [b]Away, crypts located at least 3 cm from an ALN; over, crypts located within three crypt widths of a lymphatic nodule. [c]Significantly higher mean than means of other treatment groups: $P < 0.05$.

1994; Barnes et al, 1995). A single column of cells was counted in each complete mid-axially sectioned crypt from the base up each side to the mouth of the crypt. The recorded parameters were the following: total height from the base to the mouth of the crypt in number of cells (CH), total number of darkly stained PCNA-positive cells per crypt, and position of each positively stained cell along the crypt column.

### Scoring of apoptosis

Apoptosis was scored on haematoxylin and eosin-stained, complete mid-axially sectioned colon crypts, as described previously (Potten et al, 1992), using different sections from the same tissue blocks used in assessing crypt cell proliferation. At least 30 crypts away from an ALN and 15 crypts over the distal ALN per rat were divided along the long axis into two half-crypts, which were scored separately for each of six rats per treatment group (i.e. at least 360 half-crypts away ALN and 180 half-crypts over ALN). Each apoptotic event was identified using the established morphological features of nuclear marginalization of chromatin, condensation of the cytoplasm, cell shrinkage and membrane blebbing, and final fragmentation of the cell into apoptotic bodies (Potten, 1992) using a light microscope (Figure 1). The position of each apoptotic event in number of cells from the base of the crypt was recorded. Apoptotic bodies were occasionally seen engulfed by neighbouring cells or extruded into the crypt lumen. If several apoptotic bodies were grouped around a single cell position, the cluster was recorded as a single apoptotic event. In addition, if a clear distinction could not be made between an apoptotic cell and an invading intraepithelial lymphocyte, the figure was not recorded. Only apoptotic cells within the crypt length and not at the lumenal surface between crypts, where considerable cell death occurs, were included in this assessment.

In addition, two techniques for identifying apoptotic cells were compared for general agreement. One set of slide-mounted colon tissue sections from the saline, DMH vehicle control group was stained with haematoxylin and eosin and apoptotic cells were identified by morphological characteristics. Representative examples of apoptosis are shown in Figure 1. A second set of slides was used for digoxigenin labelling and direct immunoperoxidase detection of 3′-OH cut DNA ends that usually occur during the apoptotic process. The in situ detection was performed according to the manufacturer's instructions (Apoptag kit, Oncor).

### Statistical analysis

All statistical analyses were performed using either SAS (SAS Institute, Cary, IL, USA) or PRISM (GraphPad Software, San Diego, CA, USA) statistical software. Unless otherwise stated, statistical significance was accepted when $P < 0.05$. Multiway analysis of variance (ANOVA) and Student–Newman–Keuls (SNK) multiple range tests were performed for comparison of the effects of the NSAID treatment group and location over an ALN or away from an ALN on cell proliferative and apoptotic indices within colon crypts. Student's *t*-test was used for comparison of the two methods of apoptosis assessment.

Plots of either proliferative or apoptotic cells exhibited Poisson distributions as determined by the Kolmogorov–Smirnov goodness-of-fit test. However, the square root transformed data did not differ from a normal distribution, so parametric statistical comparisons of PCNA-stained cells per crypt and crypt apoptotic index were performed on the transformed data. Crypt height was found to be normally distributed and thus no transformation was necessary. *t*-tests between the mean cell position of the transformed frequency distributions of proliferative or apoptotic cell along the length of the colon crypt were used as a measure of central tendency for determining the effect of DMH treatment and/or location over or away from the distal ALN on the intracrypt distribution of proliferative or apoptotic cells. For simplicity of presentation, these frequency distributions were smoothed over five consecutive cell positions along the length of the crypt in Figure 2.

**Table 2** Statistical comparison of crypt apoptotic indices and distribution of apoptosis in normal rat colon crypts both over an ALN and away from an ALN using identification of apoptotic cells by morphological criteria vs identification by in situ detection of digoxigenin-labelled 3′-OH DNA ends

| | Apoptotic cells/ midaxial crypt section[a] | | Distribution Means[b] | |
|---|---|---|---|---|
| Detection Method | Away ALN | Over ALN[c] | Away ALN | Over ALN[d] |
| Morphology[e] | 0.186 | 0.684 | 14.39 ± 3.03 | 21.12 ± 1.47 |
| in situ DNA end-labelling | 0.075 | 0.427 | 15.79 ± 5.17 | 21.23 ± 4.59 |

[a]The mean number of apoptotic cells per midaxial crypt section from six rats per group and at least 60 half-crypts per rat away from the ALN, and 30 half-crypts per rat over the ALN. [b]The mean of the distribution of apoptotic cells by cell position (± 95% CI) from the crypt base. [c]Over the ALN significantly greater than away from the ALN: $P < 0.05$. [d]Over the ALN significantly greater than away from the ALN: $P < 0.05$. [e]Use of morphological criteria on H&E-stained tissue sections identified significantly more apoptotic cells per midaxial crypt section than did in situ DNA end-labelling: $P < 0.05$.

© Cancer Research Campaign 1998

### RESULTS

### Proliferative parameters

The number of PCNA positively stained cells per midaxial crypt section was evaluated in each colon at the end of an 8-week initiation period as a surrogate end point biomarker (or biomarker) for risk of colon cancer development (Table 1). Two-way ANOVA evaluating the influence of location of crypts either over or away from the ALN and the influence of NSAID treatment on cell proliferation in DMH-treated rats revealed that both variables independently and significantly increased the number of proliferating cells per crypt, with a significant interaction between the two variables. Subsequent one-way ANOVA demonstrated that the interaction was due to the significant increase in proliferating cells per crypt in the aspirin and salicylate treatment groups selectively in crypts over the ALN. There were no significant changes in proliferative parameters due to indomethacin, nabumetone or $dmPGE_2$.

### Effect of DMH and NSAID intervention on total cyclooxygenase activity

A radioimmunoassay for $PGE_2$ production from radiolabelled arachidonic acid was performed on colon tissue from each experimental rat to determine if administration of PGHS inhibitors, under the current protocol, caused any long-term (i.e. several days) alteration in colon mucosal cyclo-oxygenase activity. Two-way ANOVA showed that there were no significant differences in $PGE_2$ production due to DMH treatment 3 days after the last dose of DMH [50.75 ± 18.45 (DMH) vs 41.80 ± 14.65 (no DMH) pg $PGE_2$ $mg^{-1}$ colon tissue $min^{-1}$ ± 95% confidence interval] or due to the different drug treatments 4 days after the last dose of the drug (data not shown).

### Comparison of two methods for identifying apoptotic cells

A comparison between apoptotic counts within the crypt as assessed by morphology and as assessed by in situ end-labelling of the fragmented DNA ends was conducted on colon tissue sections from the DMH vehicle/saline control group. There were quantitative differences in the apoptotic index as assessed by these two methods (Table 2). Scoring by the morphological criteria gave a higher apoptotic index than the DNA end-labelling method. However, statistical analyses showed that there was no significant difference in the mean of the spatial frequency distribution of apoptotic cells within the crypt due to the method of detection. Both techniques indicated that the number of apoptotic cells per midaxial crypt section and the mean of the frequency distribution of apoptotic cells were higher in crypts located over the ALN than in crypts away from the ALN.

The results of the morphological index were approximately that of the estimated normal rate of apoptosis in mouse colon epithelial cells (0.2 apoptotic bodies/midaxial crypt section; Potten, 1992). The DNA end-labelling method may identify fewer apoptotic cells than morphology because this technique: (a) exhibits marked variability of results; (b) is highly dependent on the conditions of the labelling procedure; and (c) appears to be difficult to use in the intestine (Que and Gores, 1996). Owing to the greater accuracy and reliability of morphological criteria, this method was used for identification of apoptotic cells for the quantitative apoptotic data presented in the remainder of this report.

### Quantitation of crypt apoptosis as influenced by DMH, NSAIDs or location in the colon

The mean number of apoptotic cells per midaxial colon crypt section in each treatment group were compared by three way ANOVA for differences due to the variables of: (a) DMH treatment; (b) location in colon (over or away from the ALN); and (c) NSAID treatment. DMH treatment did not significantly affect the occurrence of apoptosis in colon epithelial cells as assessed 3 days after the last DMH dose (data not shown), but the mean apoptotic index was significantly higher in crypts located over the distal ALN (0.754 apoptotic cells/crypt section) than in crypts located away from the distal ALN (0.219 apoptotic cells/crypt section) as shown in Table 3. There was no significant difference between the apoptotic indices of the DMH/NSAID treatment groups away from the ALN. However, over the ALN the mean apoptotic index of the salicylate treated group was significantly higher than in all other treatment groups ($P < 0.001$).

### Correlation of apoptosis and proliferation

Linear regression was used to test for correlation between the crypt apoptotic index and the cell proliferative index in crypts of individual treatment groups both with and without DMH and in crypts located over and away from the distal ALN. There were no significant correlations by linear regression analyses between cell birth (proliferation) and cell death (apoptosis) within crypts (data not shown). This lack of correlation may be due to the assessment of apoptotic cells only within the crypt column. Considerable cell death occurs at the luminal surface of the colon epithelium and apoptotic cells on the luminal surface were not assessed.

**Table 3** Statistical analyses of the mean number of apoptotic cells per H&E-stained midaxial colon crypt section in DMH-treated rats

| | Apoptotic cells/midaxial crypt section[a] | | | | | | Row means |
|---|---|---|---|---|---|---|---|
| | Saline | Aspirin | Salicylate | Indomethacin | Nabumetone | $dmPGE_2$ | |
| Away ALN[b] | 0.220 | 0.218 | 0.222 | 0.203 | 0.207 | 0.247 | 0.219 |
| Over ALN[c] | 0.738 | 0.783 | 0.973[d] | 0.636 | 0.674 | 0.717 | 0.754 |

[a]Mean number of morphologically apoptotic epithelial cells along the crypt column length from six untreated control rats per group and at least 60 half-crypts per rat away from the ALN, and 30 half-crypts per rat over the ALN. [b]Away ALN, at least 3 cm from a lymphatic nodule; over ALN, within three crypt widths of a lymphatic nodule. [c]Over the ALN significantly greater value than away from the ALN: $P < 0.05$. [d]Salicylate group was significantly different from all other groups: $P < 0.05$.

© Cancer Research Campaign 1998

**Table 4** Mean (± 95% confidence intervals) position, in number of cells from the crypt base, of the frequency distributions of: PCNA-positive proliferative cells; morphologically identified apoptotic cells; and crypt height in the descending colon of rats

| Location | DMH[a] | n[b] | Proliferative cells | Apoptotic cells | Crypt height |
|---|---|---|---|---|---|
| Away ALN | – | 717 | 7.34 ± 0.26 | 15.73 ± 1.29 | 30.10 ± 0.22 |
| | + | 440 | [d]9.43 ± 0.40 | [d]14.00 ± 1.22 | [d]32.30 ± 0.30 |
| Over ALN[c] | – | 340 | 11.26 ± 0.40 | 21.65 ± 1.40 | 38.32 ± 0.46 |
| | + | 308 | [d]13.46 ± 0.43 | [d]19.54 ± 1.41 | [e]41.92 ± 0.54 |

[a](–) DMH vehicle (+) DMH (12 mg base per kg body weight) s.c. once a week for 8 weeks. [b]Number of crypts evaluated for proliferative cells, apoptotic cells, and crypt height. [c]Mean position of proliferative cells and apoptotic cells from the crypt base, and the mean colon crypt height were significantly greater over the ALN than away from the ALN, both with and without DMH. [d]Significant change from the non-DMH group. Square root transformation was used to normalize data, then the means were compared using the unpaired *t*-test. Untransformed data are presented in the table. [e]Significant change from the non-DMH group when compared using the unpaired *t*-test.

### Distribution of proliferating cells and apoptotic cells in colon crypts

There were no significant differences between NSAID treatment groups in the distribution of PCNA-positive cells, the distribution of apoptotic cells along the length of the crypt, or in crypt height in number of cells (data not shown). Therefore, distributional data from all treatment groups were combined for analyses of the effects of crypt location (over vs away from ALN) and DMH treatment on the frequency distributions of proliferating cells and of apoptotic cells along the crypt length, and of mean crypt height (Figure 2). Proliferative cells were located mainly in the lower one-third to two-thirds of the crypt, whereas apoptotic cells were found at every cell position along the length of the crypt. Comparison of the means of the distributions of proliferation, apoptosis and crypt height using the *t*-test showed that the means of all three distributions were higher in crypts located over the ALN than in crypts located away from the ALN whether or not the rats were exposed to DMH (Table 4). DMH treatment resulted in: (a) a significant shift in the mean position of proliferative cells towards the top of the crypt; (b) a significant shift in the mean position of apoptotic cells towards the base of the crypt; and (c) a significant increase in the mean crypt height (Table 4). The magnitude of this DMH effect on the mean position of proliferative and apoptotic cells was similar in crypts over and away from ALN, possibly indicating a similar response to the carcinogen in both regions of the colon.

### DISCUSSION

### NSAIDs and ALN on cell proliferation

Colon crypt epithelial cell proliferation was evaluated at the end of an 8-week initiation period in this rat colon cancer model, specifically 4 days after the last NSAID dose and 3 days after the last DMH dose. This recovery time was necessary to allow the epithelium to reach a steady state after DMH injection (Wright and Alison, 1984; Deschner, 1987; Cameron et al, 1990), and to enable the evaluation of longer lasting rather than immediate effects of the other drugs. Given the relatively short half-lives of the drugs administered (Insel, 1996) and the lack of any significant difference in total cyclo-oxygenase activity between the treatment groups, any changes in proliferation may have been due to a long-term influence of the NSAID rather than to a short-term NSAID suppression of prostaglandin synthesis. However, 4 days after the last dose there was no significant change in crypt proliferation due to indomethacin, nabumetone or dm$PGE_2$ treatment when compared with the saline/DMH control group. Thus, it is probable that these drugs do not have a long-lasting influence on colon epithelial cell proliferation. There were significant increases in the mean number of proliferating cells per crypt 4 days after salicylate or aspirin treatment, which occurred selectively in crypts located over the ALN, although the saline/DMH control group had a spuriously low PCNA index. The relevance of these findings is discussed below in relation to altered apoptosis within the same colon location and treatment groups. Finally, we would like to point out that although we have used strict counting criteria to avoid excessive proliferative counts, there are potential limitations to using PCNA as a proliferative marker, such as the long half-life of PCNA and its expression during DNA repair, leading to the labelling of non-proliferative cells (Hall et al, 1990; Risio et al, 1994).

Crypt cell proliferation was significantly higher following DMH treatment, and also significantly higher in crypts located over an ALN than in crypts located away from an ALN. These findings were expected based on previous reports (Hardman and Cameron, 1994; Barnes et al, 1995). As there is a strong correlation between sites of lymphatic nodules and sites of high incidence of colon and rectal tumours in both rodents (Hammann et al, 1992; Cameron et al, 1996) and humans (Shamsuddin et al, 1982; Glick et al, 1988; Hillon et al, 1990), it seems important to evaluate the ability of potential chemopreventive agents to modulate surrogate end point biomarkers in colon crypts in the region of the ALN if we are to be able to predict the efficacy of the agent at preventing colon cancer.

### Alterations in crypt apoptotic index

The transformation of colorectal epithelium to carcinoma has been associated with a progressive inhibition of apoptosis (Bedi et al, 1995; Payne et al, 1995) and an increase in the proliferative– apoptotic cell ratio (Koike, 1996). Apoptosis within the crypt column is thought to eliminate crypt cells containing random and/or induced genetic defects (Potten, 1992). Perturbation of the normal pattern of apoptosis may, therefore, serve as an early surrogate end point biomarker of both carcinogen exposure and disease progression. We have for the first time used alteration in the number and the distribution of apoptotic cells within rat colon crypts to indicate both early preneoplastic changes and the efficacy of chemopreventive interventions.

 © Cancer Research Campaign 1998

The findings reported here reveal an overall significant increase in cell proliferation in the midaxially sectioned colon crypts 3 days after repeated carcinogen exposure without a significant persistent increase in the apoptotic index. Increased proliferation without increased cell death might be expected to result in an accumulation of cells in the crypt column height, which was indeed observed. However, conclusions cannot be drawn from the present data on altered crypt cell volume. It is likely that the observations reported here of an overall increase in proliferation and decrease apoptotic cell death within the crypt column of midaxially sectioned colon crypts reflect a perturbation from the normal crypt homeostasis after repeated carcinogen exposures.

Treatment with aspirin or sodium salicylate resulted in increased proliferation and a higher apoptotic index (significant for salicylate only) selectively in crypts over the distal ALN. Considering the fact that salicylic acid, the metabolite of aspirin, has a much longer biological half-life than does aspirin (Insel, 1996), and that evaluations were performed 4 days after the last aspirin dose, one can conjecture that the observed aspirin effect may actually be due to exposure to salicylate rather than as a direct action of aspirin itself. In support of this hypothesis, in vitro studies report that salicylate treatment (Elder et al, 1996) but not aspirin treatment (Shiff et al, 1996) induced apoptotic cell death in cultured epithelial cells. Increased epithelial cell turnover rate (apoptosis and proliferation) after carcinogen exposure may be indicative of self-screening, i.e. identification and elimination of cells with irreparable genetic damage, resulting in compensatory increased cell proliferation. Thus, one potential cancer chemopreventive mechanism of action for aspirin may be to increase death of damaged cells via salicylate, specifically in the crypts located over the distal ALN, which are at high risk for developing cancer (Hardman and Cameron, 1994; Cameron et al, 1996). Indeed, induction of apoptosis has been proposed as the primary mechanism of action of the NSAID sulindac and its sulphide and sulphone derivatives in countering familial adenomatous polyposis (Pasricha et al, 1995; Shiff et al, 1995).

### Distribution of proliferative and apoptotic cells within colon crypts

DMH appears to effect a long-term (i.e. at least 3 days) coordinated, spatial alteration in the distribution of cell birth and cell death within the crypt. The significant reduction in the relative distance between the peaks of the distribution of cell proliferation and cell death after DMH treatment may be indicative of greater apoptotic cell death of the less differentiated progenitors of epithelial cells that are found deeper in the crypt. Upon repeated carcinogen exposure, crypt stem cells are likely to sustain and accumulate genetic damage, but are hypothesized to be unable to eliminate themselves by undergoing apoptosis due to high levels of Bcl-2 expression (Merritt et al, 1995). The progeny of stem cells, however, lose this high level of Bcl-2 expression (Merritt et al, 1995) and gain the capacity for self-screening and subsequent elimination of damaged cells through induction of apoptosis. With a greater number of progeny cells dying earlier in the differentiation process, fewer cells remain to repopulate the crypt. Thus, the remaining cells must undergo a greater number of divisions to maintain the normal number of crypt cells. The result is a delay in terminal cell differentiation, the occurrence of proliferating cells higher in the crypt column, and a significant expansion of the crypt proliferative zone after carcinogen exposure (Deschner and Maskens, 1982; Wright and Alison, 1984). Eventually, some cells with genetic damage are likely to escape this self-screening mechanism, leading to proliferation of carcinogen-initiated cells and the eventual development of intraepithelial neoplasms. Aspirin or salicylate may somehow enhance the self-screening capacity and subsequently the elimination of damaged cells.

### CONCLUSION

Aspirin and salicylate are hypothesized to enhance self-screening of DMH-initiated colon epithelial cells selectively in crypts located over the distal aggregate of lymphoid nodules, a site at high risk for adenocarcinoma development, suggesting a possible anti-cancer mechanism of action for aspirin. In the present model of DMH-induced rat colon cancer, administration of indomethacin and nabumetone did not alter surrogate end point biomarkers when evaluated 4 days after the last NSAID dose, suggesting that these NSAIDs may not have the same chemopreventive ability as aspirin. Perturbation of the normal colon crypt apoptotic index and the normal distribution of apoptotic cells within the crypt were successfully used as surrogate biomarkers of colon carcinogen exposure. Given the importance of both cell birth and cell death in the carcinogenic process, colon crypt epithelial cell apoptosis in conjunction with cell proliferation should be evaluated in studies of colon carcinogenesis.

### ACKNOWLEDGEMENTS

This work was supported by a VA Merit Review Award (VA-93-001) and a NIH Research Award (P30 CA 54174).

### ABBREVIATIONS

ALN, aggregate of lymphoid nodules; ANOVA, analysis of variance; CH, crypt height; DMH, 1,2-dimethylhydrazine; $dmPGE_2$, 16,16-dimethyl prostaglandin $E_2$; NSAID, non-steroidal anti-inflammatory drug; PCNA, proliferating cell nuclear antigen; $PGE_2$, prostaglandin $E_2$; PGHS, prostaglandin endoperoxide H synthase; SNK, Student–Newman–Keuls; TdT, terminal deoxynucleotidal transferase.

### REFERENCES

Barnes CJ, Lee M, Hardman WE and Cameron IL (1995) Aspirin, age, and proximity to lymphoid nodules influence cell proliferation parameters in rat colonic crypts. *Cell Prolif* **28**: 59–71

Bedi A, Pasricha PJ, Akhtar AJ, Barber JP, Bedi GC, Giardiello FM, Zehnbauer BA, Hamilton SR and Jones RJ (1995) Inhibition of apoptosis during development of colorectal cancer. *Cancer Res* **55**: 1811–1816

Bostick R, Fosdick L, Overn P, Lillemoe T, Wood J, Grandits G, Grambsch P, Forster C, Bartz L, Shields H and Potter J (1994) Superiority of the proliferating cell nuclear antigen vs the bromodeoxyuridine technique for measuring epithelial cell proliferation in humans. *Proc Am Assoc Cancer Res* **35**: 633

Cameron IL, Ord VA, Hunter KE and Heitman DW (1990) Colon carcinogenesis: modulation of progression. In *Colon Cancer Cells*. Moyer MP and Poste G (eds), pp. 63–84. Academic Press: Orlando, FL

Cameron IL, Garza J and Hardman WE (1996) Distribution of lymphoid nodules, aberrant crypt foci and tumours in the colon of carcinogen-treated rats. *Br J Cancer* **73**: 893–898

Craven PA and Derubertis FR (1992) Effects of aspirin on 1,2-dimethylhydrazine-induced colonic carcinogenesis. *Carcinogenesis* **13**: 541–546

Deschner EE (1987) Cell turnover and colon tumour development. *Prev Med* **16**: 580–585

© Cancer Research Campaign 1998

Deschner EE and Maskens AP (1982) Significance of the labelling index and labelling distribution as kinetic parameters in colorectal mucosa of cancer patients and DMH treated animals. *Cancer* **50**: 1136–1141

Dubois RN, Radhika A, Reddy BS and Entingh AJ (1996) Increased cyclooxygenase-2 levels in carcinogen-induced rat colonic tumours. *Gastroenterology* **110**: 1259–1262

Earnest DL, Hixson LJ and Alberts DS (1992) Piroxicam and other cyclooxygenase inhibitors: potential for cancer chemoprevention. *J Cell Biochem* **161**(suppl): 156–166

Eberhart CE, Coffey RJ, Radhika A, Giardiello FM, Ferrenbach S and Dubois RN (1994) Up-regulation of cyclooxygenase 2 gene expression in human colorectal adenomas and adenocarcinomas. *Gastroenterology* **107**: 1183–1188

Elder DJE, Hague A, Hicks DJ and Paraskeva C (1996) Differential growth inhibition by the aspirin metabolite salicylate in human colorectal tumour cell lines: enhanced apoptosis in carcinoma and *in vitro*-transformed adenoma relative to adenoma cell lines. *Cancer Res* **56**: 2273–2276

Giardiello FM, Offerhaus GJA and Dubois RN (1995) The role of non-steroidal anti-inflammatory drugs in colorectal cancer prevention. *Eur J Cancer* **31A**: 1071–1076·

Giovannucci E, Egan KM, Hunter DJ, Stampfer MJ, Colditz GA, Willett WC and Speizer FE (1995) Aspirin and the risk of colorectal cancer in women. *N Engl J Med* **333**: 609–614

Glick SN, Teplick SK and Ross WM (1988) Colonic lymphoid follicles associated with colonic neoplasms. *Radiology* **168**: 603–607

Goodwin JS (1981) Prostaglandins and host defense in cancer. *Med Clin N Am* **65**: 829–844

Hall PA, Levison DA, Woods AL, Yu CC-W, Kellock DB, Watkins JA, Barnes DM, Gillett CE, Camplejohn R, Dover R, Waseem NH and Lane DP (1990) Proliferating cell nuclear antigen (PCNA) immunolocalization in paraffin sections: an index of cell proliferation with evidence of deregulated expression in some neoplasms. *J Pathol* **162**: 285–294

Hammann A, Arveux P and Martin M (1992) Effect of gut-associated lymphoid tissue on cellular proliferation in proximal and distal colon of the rat. *Dig Dis Sci* **37**: 1099–1104

Hardman WE and Cameron IL (1994) Colonic crypts located over lymphoid nodules of 1,2-dimethylhydrazine-treated rats are hyperplastic and at high risk of forming adenocarcinomas. *Carcinogenesis* **15**: 2353–2361

Hillon P, Martin MS, Piard F and Jacquot JF (1990) Relationship between adenomas and colorectal-associated lymphoid tissue in familial polyposis coli. *Dig Dis Sci* **35**: 1307–1308

Insel PA (1996) Analgesic-antipyretic and antiinflammatory agents and drugs employed in the treatment of gout. In *Goodman and Gilman's The Pharmacological Basis of Therapeutics*, 9th edn. Hardman JG, Limbird LE, Molinoff PB, Ruddon RW and Gilman AG (eds), pp. 617–658. McGraw-Hill: New York.

Kargman SL, O'Neill GP, Vickers PJ, Evans JF, Mancini JA and Jothy S (1995) Expression of prostaglandin G/H synthase-1 and -2 protein in human colon cancer. *Cancer Res* **55**: 2556–2559

Koike M (1996) Significance of spontaneous apoptosis during colorectal tumorigenesis. *J Surg Oncol* **62**: 97–108

Laneuville O, Breuer DK, Dewitt DL, Hla T, Funk CD and Smith WL (1994) Differential inhibition of human prostaglandin endoperoxide synthases-1 and –2 by nonsteroidal anti-inflammatory drugs. *J Pharm Exp Ther* **271**: 927–934

Lee M and Feldman M (1992) Nonessential role of leukotrienes as mediators of acute gastric mucosal injury induced by aspirin in rats. *Dig Dis Sci* **37**: 1282–1287

Marnett LJ (1992) Aspirin and the potential role of prostaglandins in colon cancer. *Cancer Res* **52**: 5575–5589

Meade EA, Smith WL and Dewitt DL (1993) Differential inhibition of prostaglandin endoperoxide synthase (cyclooxygenase) isozymes by aspirin and other non-steroidal anti-inflammatory drugs. *J Biol Chem* **268**: 6610–6614

Merritt AJ, Potten CS, Watson AJ, Loh DY, Nakayama K and Hickman JA (1995) Differential expression of bcl-2 in intestinal epithelia. Correlation with attenuation of apoptosis in colonic crypts and the incidence of colonic neoplasia. *J Cell Sci* **108**: 2261–2271

Pasricha PJ, Bedi A, O'Connor K, Rashid A, Akhtar AJ, Zahurak ML, Piantadosi S, Hamilton SR and Giardiello FM (1995) The effects of sulindac on colorectal proliferation and apoptosis in familial adenomatous polyposis. *Gastroenterology* **109**: 994–998

Payne CM, Bernstein H, Bernstein C and Garewal H (1995) Role of apoptosis in biology and pathology: resistance to apoptosis in colon carcinogenesis. *Ultrastruct Pathol* **19**: 221–248

Potten CS (1992) The significance of spontaneous and induced apoptosis in the gastrointestinal tract of mice. *Cancer Metast Rev* **11**: 179–195

Potten CS, Li Y, O'Connor PJ and Winton DJ (1992) A possible explanation for the differential cancer incidence in the intestine, based on distribution of the cytotoxic effects of carcinogens in the murine large bowel. *Carcinogenesis* **13**: 2305–2312

Pugh S and Thomas GAO (1994) Patients with adenomatous polyps and carcinomas have increased colonic mucosal prostaglandin $E_2$. *Gut* **35**: 675–678

Qiao L, Kozoni V, Tsioulias GJ, Koutsos MI, Hanif R, Shiff SJ and Rigas B (1995) Selected eicosanoids increase the proliferation rate of human colon carcinoma cell lines and mouse colonocytes in vivo. *Biochim Biophys Acta* **1258**: 215–223

Que FG and Gores GJ (1996) Cell death by apoptosis: basic concepts and disease relevance for the gastroenterologist. *Gastroenterology* **110**: 1238–1243

Reddy BS, Rao CV, Rivenson A and Kelloff G (1993) Inhibitory effect of aspirin on azoxymethane-induced colon carcinogenesis in F344 rats. *Carcinogenesis* **14**: 1493–1497

Redfern JS, Lee E, and Feldman M (1987) Effect of indomethacin on gastric muscosal prostaglandins in humans. *Gastroenterology* **92**: 969–977

Risio M (1994) methodological aspects of using immunohistochemical cell proliferation biomarkers in colorectal carcinoma chemoprevention. *J Cell Biochem* **19**: (suppl.) 61–67

Risio M, Candelaresi G and Rossini FP (1993) Bromodeoxyuridine uptake and proliferating cell nuclear antigen expression throughout the colorectal tumor sequence. *Cancer Epid Biomark Prev* **2**: 363–367

Shamsuddin AM, Phelps PC and Trump BF (1982) Human large intestinal epithelium: Light microscopy, histochemistry, and ultrastructure. *Hum Pathol* **13**: 790–803

Shiff SJ, Qiao L, Tsai L and Rigas B (1995) Sulindac sulfide, an aspirin-like compound, inhibits proliferation, causes cell cycle quiescence, and induces apoptosis in HT-29 colon adenocarcinoma cells. *J Clin Invest* **96**: 491–503

Smith WL and Dewitt DL (1996) Prostaglandin endoperoxide synthases-1 and -2. *Adv Immunol* **62**: 167–215

Thun MJ (1994) Aspirin, NSAIDs, and digestive tract cancers. *Cancer Metast Rev* **13**: 269–277

Wright N and Alison M (1984) Cell proliferation in gastrointestinal carcinogenesis. In *The Biology of Epithelial Cell Populations*, Vol 2. Oxford University Press: New York

© Cancer Research Campaign 1998


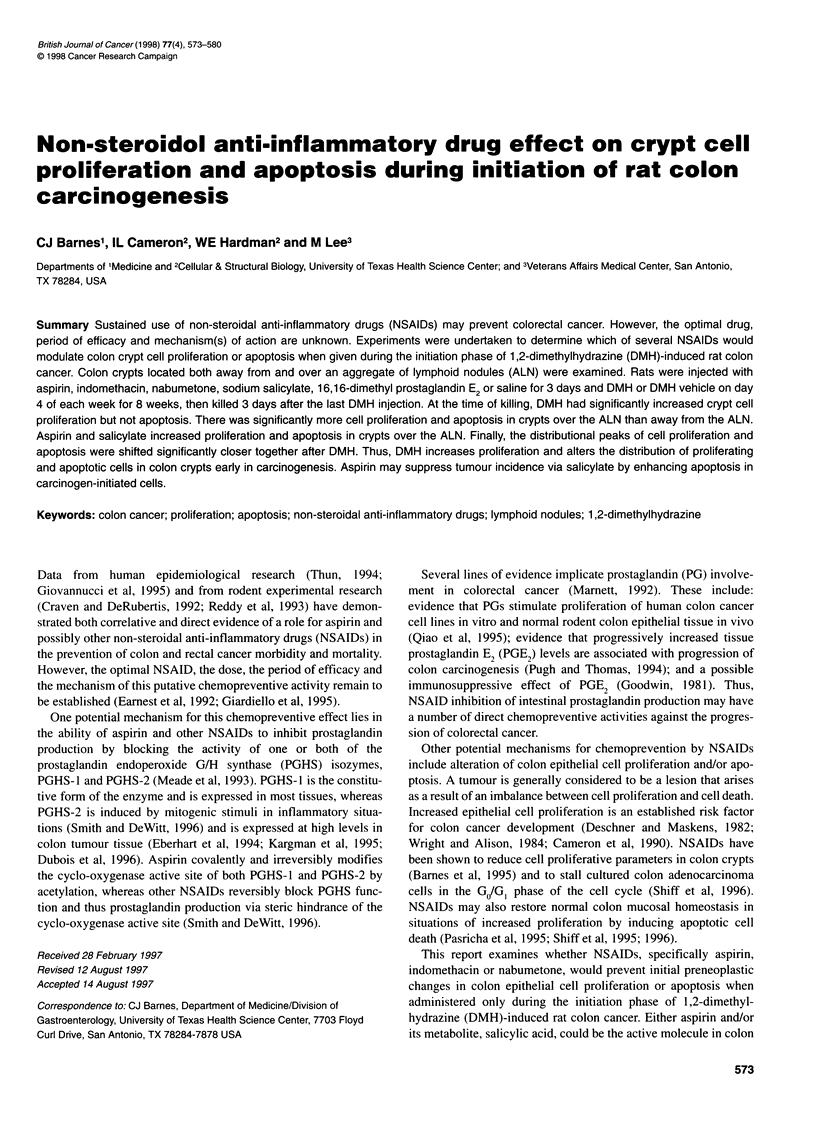

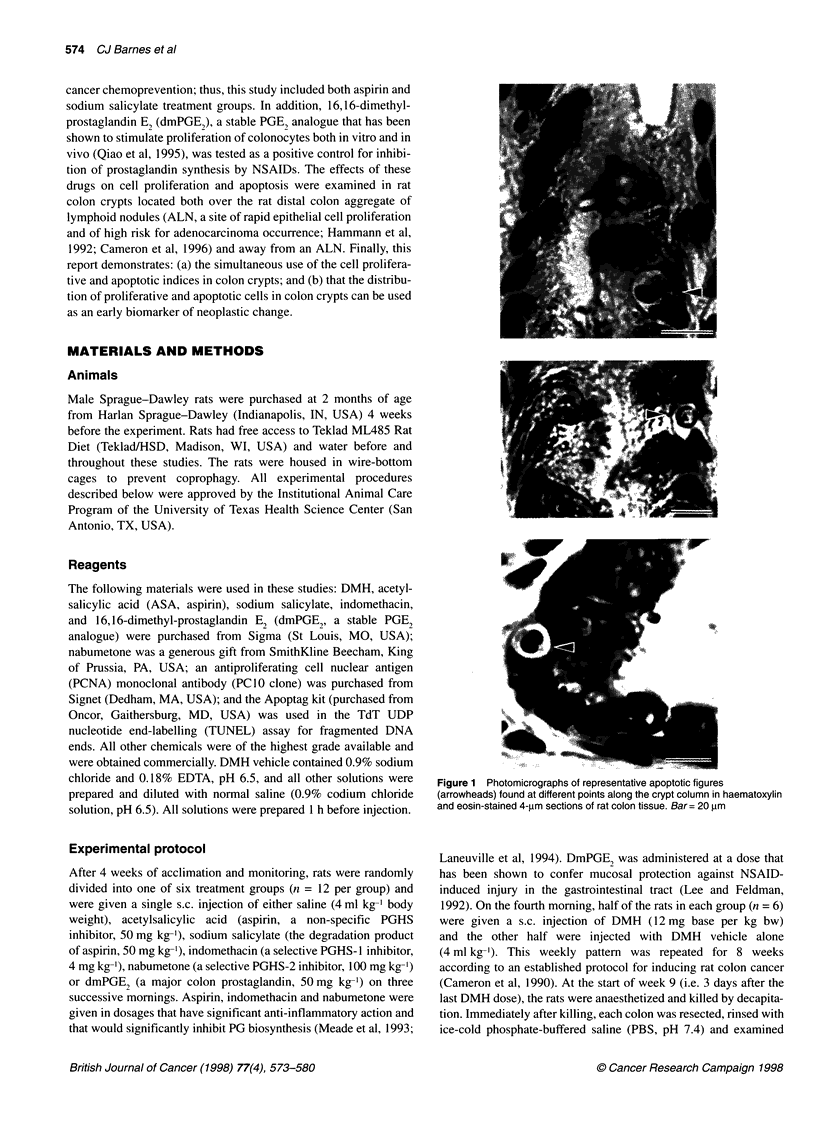

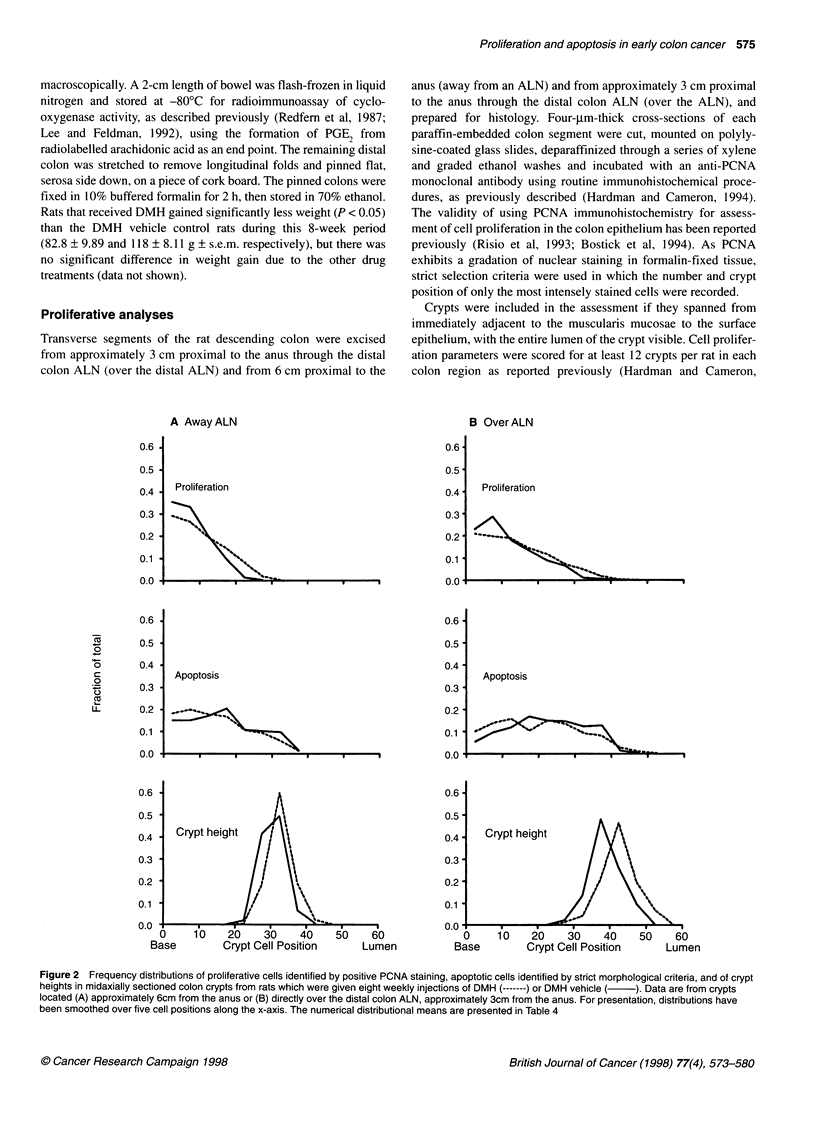

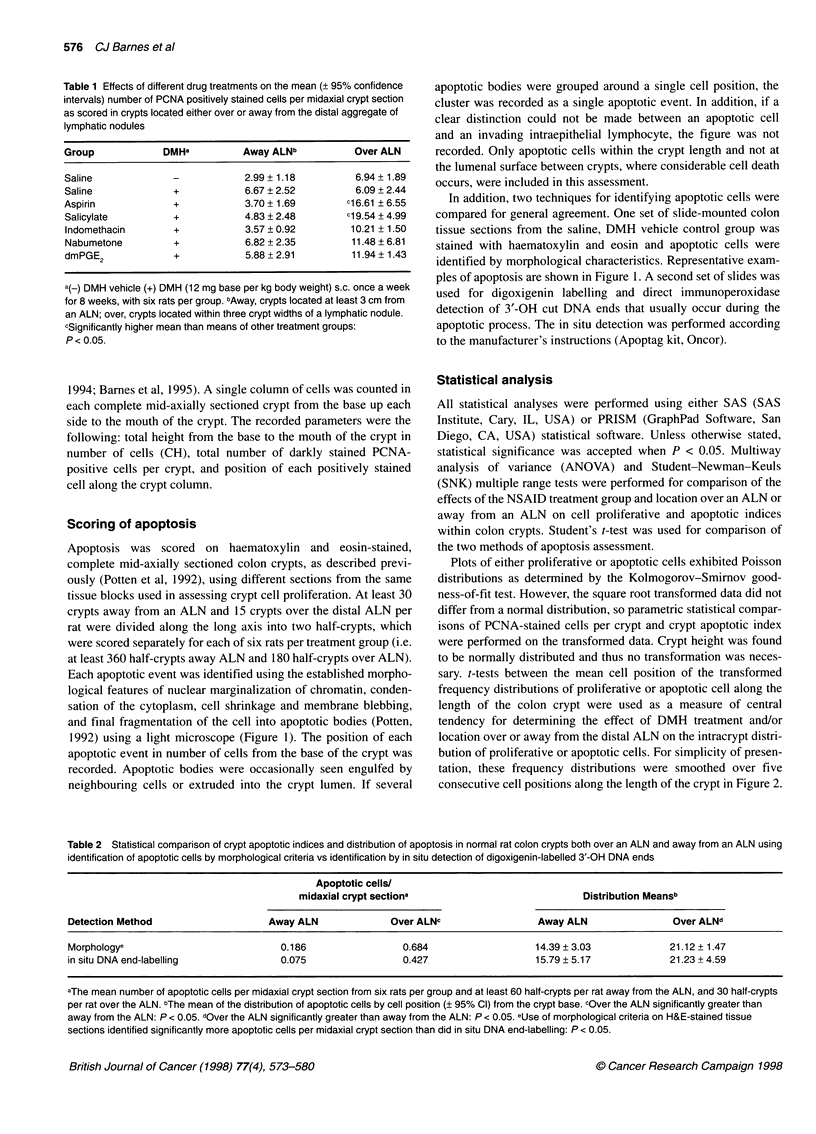

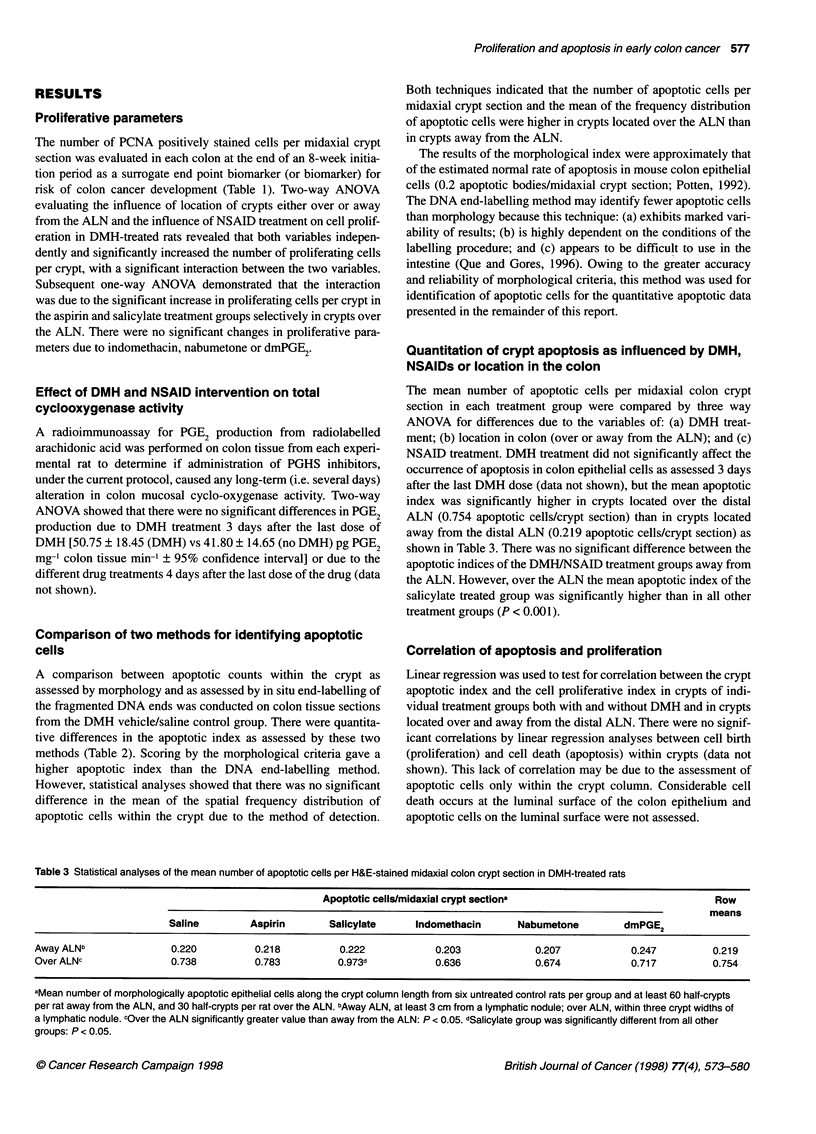

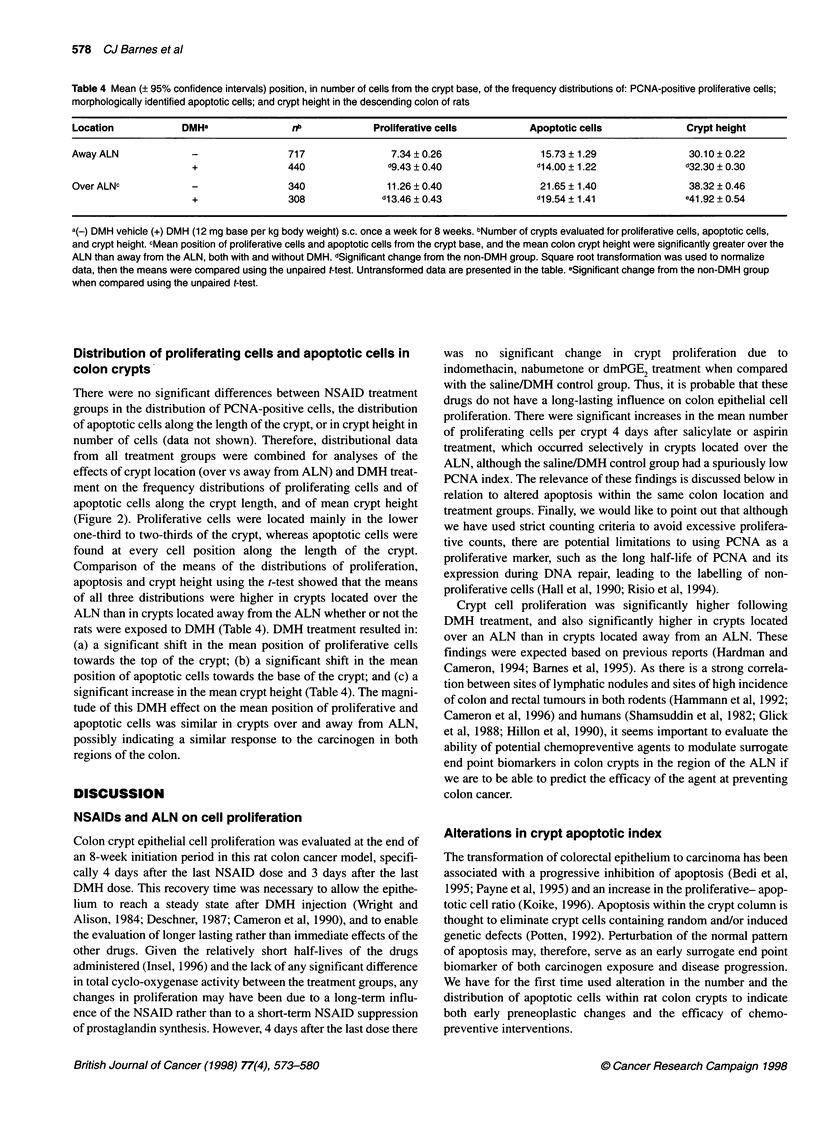

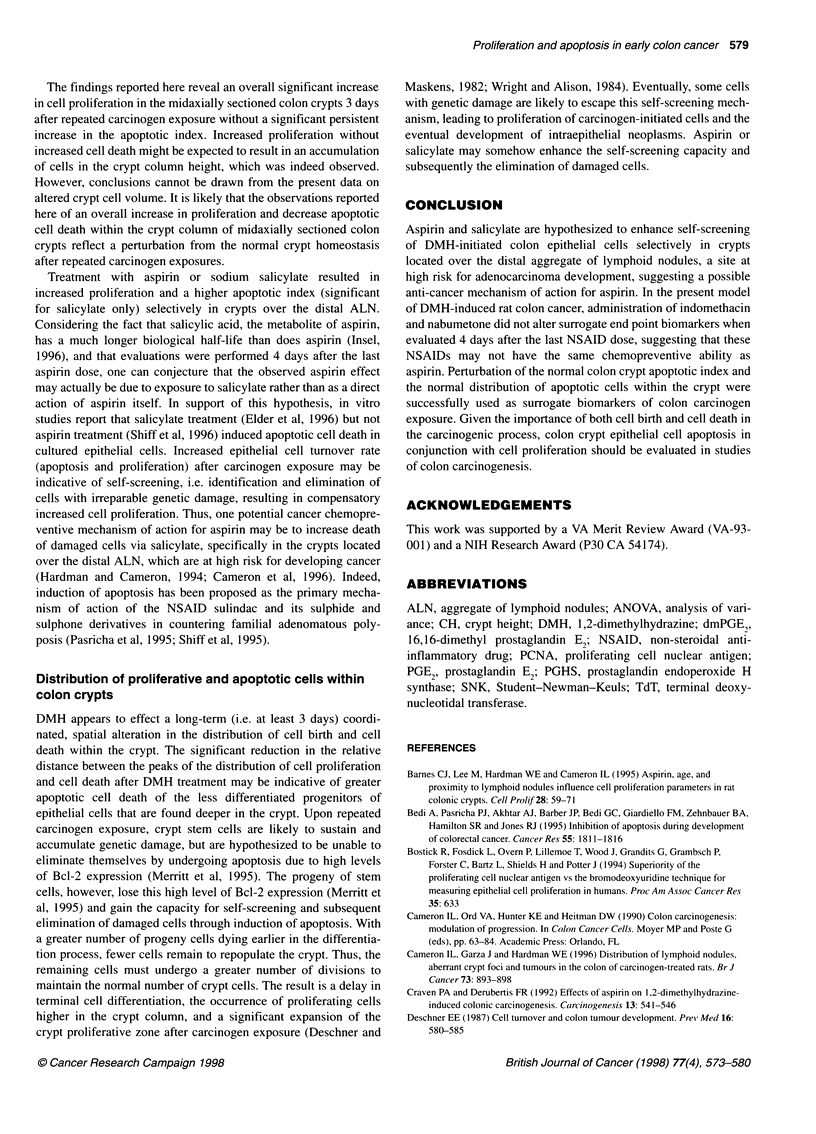

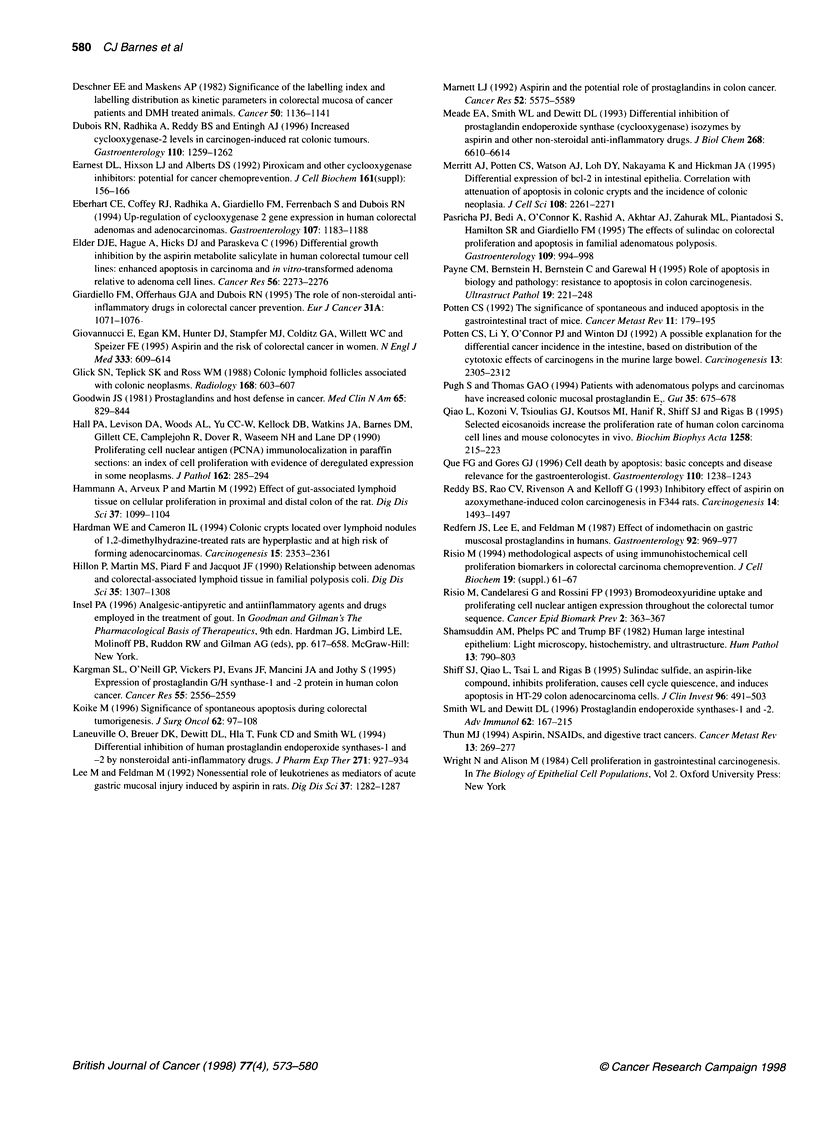


## References

[OCR_00801] Barnes C. J., Lee M., Hardman W. E., Cameron I. L. (1995). Aspirin, age, and proximity to lymphoid nodules influence cell proliferation parameters in rat colonic crypts.. Cell Prolif.

[OCR_00806] Bedi A., Pasricha P. J., Akhtar A. J., Barber J. P., Bedi G. C., Giardiello F. M., Zehnbauer B. A., Hamilton S. R., Jones R. J. (1995). Inhibition of apoptosis during development of colorectal cancer.. Cancer Res.

[OCR_00820] Cameron I. L., Garza J., Hardman W. E. (1996). Distribution of lymphoid nodules, aberrant crypt foci and tumours in the colon of carcinogen-treated rats.. Br J Cancer.

[OCR_00830] Craven P. A., DeRubertis F. R. (1992). Effects of aspirin on 1,2-dimethylhydrazine-induced colonic carcinogenesis.. Carcinogenesis.

[OCR_00834] Deschner E. E. (1987). Cell turnover and colon tumor development.. Prev Med.

[OCR_00842] Deschner E. E., Maskens A. P. (1982). Significance of the labeling index and labeling distribution as kinetic parameters in colorectal mucosa of cancer patients and DMH treated animals.. Cancer.

[OCR_00847] DuBois R. N., Radhika A., Reddy B. S., Entingh A. J. (1996). Increased cyclooxygenase-2 levels in carcinogen-induced rat colonic tumors.. Gastroenterology.

[OCR_00852] Earnest D. L., Hixson L. J., Alberts D. S. (1992). Piroxicam and other cyclooxygenase inhibitors: potential for cancer chemoprevention.. J Cell Biochem Suppl.

[OCR_00857] Eberhart C. E., Coffey R. J., Radhika A., Giardiello F. M., Ferrenbach S., DuBois R. N. (1994). Up-regulation of cyclooxygenase 2 gene expression in human colorectal adenomas and adenocarcinomas.. Gastroenterology.

[OCR_00862] Elder D. J., Hague A., Hicks D. J., Paraskeva C. (1996). Differential growth inhibition by the aspirin metabolite salicylate in human colorectal tumor cell lines: enhanced apoptosis in carcinoma and in vitro-transformed adenoma relative to adenoma relative to adenoma cell lines.. Cancer Res.

[OCR_00868] Giardiello F. M., Offerhaus G. J., DuBois R. N. (1995). The role of nonsteroidal anti-inflammatory drugs in colorectal cancer prevention.. Eur J Cancer.

[OCR_00873] Giovannucci E., Egan K. M., Hunter D. J., Stampfer M. J., Colditz G. A., Willett W. C., Speizer F. E. (1995). Aspirin and the risk of colorectal cancer in women.. N Engl J Med.

[OCR_00878] Glick S. N., Teplick S. K., Ross W. M. (1988). Colonic lymphoid follicles associated with colonic neoplasms.. Radiology.

[OCR_00882] Goodwin J. S. (1981). Prostaglandins and host defense in cancer.. Med Clin North Am.

[OCR_00886] Hall P. A., Levison D. A., Woods A. L., Yu C. C., Kellock D. B., Watkins J. A., Barnes D. M., Gillett C. E., Camplejohn R., Dover R. (1990). Proliferating cell nuclear antigen (PCNA) immunolocalization in paraffin sections: an index of cell proliferation with evidence of deregulated expression in some neoplasms.. J Pathol.

[OCR_00895] Hammann A., Arveux P., Martin M. (1992). Effect of gut-associated lymphoid tissue on cellular proliferation in proximal and distal colon of the rat.. Dig Dis Sci.

[OCR_00900] Hardman W. E., Cameron I. L. (1994). Colonic crypts located over lymphoid nodules of 1,2-dimethylhydrazine-treated rats are hyperplastic and at high risk of forming adenocarcinomas.. Carcinogenesis.

[OCR_00905] Hillon P., Martin M. S., Piard F., Jacquot J. F. (1990). Relation between adenomas and colorectal-associated lymphoid tissue in familial polyposis coli.. Dig Dis Sci.

[OCR_00919] Kargman S. L., O'Neill G. P., Vickers P. J., Evans J. F., Mancini J. A., Jothy S. (1995). Expression of prostaglandin G/H synthase-1 and -2 protein in human colon cancer.. Cancer Res.

[OCR_00924] Koike M. (1996). Significance of spontaneous apoptosis during colorectal tumorigenesis.. J Surg Oncol.

[OCR_00928] Laneuville O., Breuer D. K., Dewitt D. L., Hla T., Funk C. D., Smith W. L. (1994). Differential inhibition of human prostaglandin endoperoxide H synthases-1 and -2 by nonsteroidal anti-inflammatory drugs.. J Pharmacol Exp Ther.

[OCR_00933] Lee M., Feldman M. (1992). Nonessential role of leukotrienes as mediators of acute gastric mucosal injury induced by aspirin in rats.. Dig Dis Sci.

[OCR_00937] Marnett L. J. (1992). Aspirin and the potential role of prostaglandins in colon cancer.. Cancer Res.

[OCR_00941] Meade E. A., Smith W. L., DeWitt D. L. (1993). Differential inhibition of prostaglandin endoperoxide synthase (cyclooxygenase) isozymes by aspirin and other non-steroidal anti-inflammatory drugs.. J Biol Chem.

[OCR_00948] Merritt A. J., Potten C. S., Watson A. J., Loh D. Y., Nakayama K., Nakayama K., Hickman J. A. (1995). Differential expression of bcl-2 in intestinal epithelia. Correlation with attenuation of apoptosis in colonic crypts and the incidence of colonic neoplasia.. J Cell Sci.

[OCR_00954] Pasricha P. J., Bedi A., O'Connor K., Rashid A., Akhtar A. J., Zahurak M. L., Piantadosi S., Hamilton S. R., Giardiello F. M. (1995). The effects of sulindac on colorectal proliferation and apoptosis in familial adenomatous polyposis.. Gastroenterology.

[OCR_00960] Payne C. M., Bernstein H., Bernstein C., Garewal H. (1995). Role of apoptosis in biology and pathology: resistance to apoptosis in colon carcinogenesis.. Ultrastruct Pathol.

[OCR_00969] Potten C. S., Li Y. Q., O'Connor P. J., Winton D. J. (1992). A possible explanation for the differential cancer incidence in the intestine, based on distribution of the cytotoxic effects of carcinogens in the murine large bowel.. Carcinogenesis.

[OCR_00965] Potten C. S. (1992). The significance of spontaneous and induced apoptosis in the gastrointestinal tract of mice.. Cancer Metastasis Rev.

[OCR_00976] Pugh S., Thomas G. A. (1994). Patients with adenomatous polyps and carcinomas have increased colonic mucosal prostaglandin E2.. Gut.

[OCR_00980] Qiao L., Kozoni V., Tsioulias G. J., Koutsos M. I., Hanif R., Shiff S. J., Rigas B. (1995). Selected eicosanoids increase the proliferation rate of human colon carcinoma cell lines and mouse colonocytes in vivo.. Biochim Biophys Acta.

[OCR_00986] Que F. G., Gores G. J. (1996). Cell death by apoptosis: basic concepts and disease relevance for the gastroenterologist.. Gastroenterology.

[OCR_00990] Reddy B. S., Rao C. V., Rivenson A., Kelloff G. (1993). Inhibitory effect of aspirin on azoxymethane-induced colon carcinogenesis in F344 rats.. Carcinogenesis.

[OCR_00995] Redfern J. S., Lee E., Feldman M. (1987). Effect of indomethacin on gastric mucosal prostaglandins in humans. Correlation with mucosal damage.. Gastroenterology.

[OCR_01004] Risio M., Candelaresi G., Rossini F. P. (1993). Bromodeoxyuridine uptake and proliferating cell nuclear antigen expression throughout the colorectal tumor sequence.. Cancer Epidemiol Biomarkers Prev.

[OCR_00999] Risio M. (1994). Methodological aspects of using immunohistochemical cell proliferation biomarkers in colorectal carcinoma chemoprevention.. J Cell Biochem Suppl.

[OCR_01009] Shamsuddin A. M., Phelps P. C., Trump B. F. (1982). Human large intestinal epithelium: light microscopy, histochemistry, and ultrastructure.. Hum Pathol.

[OCR_01014] Shiff S. J., Qiao L., Tsai L. L., Rigas B. (1995). Sulindac sulfide, an aspirin-like compound, inhibits proliferation, causes cell cycle quiescence, and induces apoptosis in HT-29 colon adenocarcinoma cells.. J Clin Invest.

[OCR_01019] Smith W. L., Dewitt D. L. (1996). Prostaglandin endoperoxide H synthases-1 and -2.. Adv Immunol.

[OCR_01023] Thun M. J. (1994). Aspirin, NSAIDs, and digestive tract cancers.. Cancer Metastasis Rev.

